# Assessing Macular Vessel Density in Iraqi Cone Dystrophy Patients Using Optical Coherence Tomography Angiography (OCTA): A Cross-Sectional Study

**DOI:** 10.7759/cureus.77455

**Published:** 2025-01-15

**Authors:** Mohammad Abbas, Ahmed S Al-Wassiti

**Affiliations:** 1 Department of Surgery, Ophthalmology Unit, Ghazi Al-Harriri for Surgical Specialities Hospital, Medical City Complex, Baghdad, IRQ; 2 Department of Surgery, University of Baghdad, College of Medicine, Baghdad, IRQ

**Keywords:** cone dystrophy, macular vessel density, non-invasive imaging, octa, retinal disorders

## Abstract

Background

Cone dystrophy is a hereditary retinopathy characterized by profound vision loss resulting from the degeneration of photoreceptors. Optical coherence tomography angiography (OCTA) is a technique that non-invasively visualizes the microvasculatures of the retina and choroid in detail. This study evaluates macular vessel density in Iraqi patients diagnosed with cone dystrophy using OCTA and makes a comparison with those of healthy controls.

Methods

The cross-sectional study was conducted at the Gazi Al-Harrir for Surgical Specialty Hospital from December 2021 to May 2024. Twenty-eight patients diagnosed with cone dystrophy and 28 healthy controls were evaluated based on measurement of best corrected visual acuity and thorough funduscopic examination. OCTA assessment of macular vessel density was performed in the central, superior, inferior, nasal, and temporal regions. All the statistical analyses were done with the software Jamovi (https://www.jamovi.org/): descriptive statistics, Shapiro-Wilk tests for normality, and independent samples t-tests.

Results

The mean central retinal thickness (CRT) was significantly lower in the cone dystrophy group (180.8 µm) compared to the control group (238.8 µm). Regarding macular vessel density, no significant differences were observed between the cone dystrophy and control groups in the central (13.4 µm vs. 17.7 µm, p = 0.234), superior (51.1 µm vs. 48.7 µm, p = 0.400), nasal (44.2 µm vs. 43.4 µm, p = 0.544), and temporal (45.1 µm vs. 45.5 µm, p = 0.202) regions. However, in the inferior region, a significant reduction in macular vessel density was observed in the control group compared to cone dystrophy patients (48.7 µm vs. 46.7 µm, p = 0.008). The Shapiro-Wilk test confirmed normal distribution for most parameters, and significant differences were identified using t-tests.

Conclusion

This study confirms that the macular vessel density is significantly reduced in Iraqi patients with cone dystrophy. OCTA proves to be a valuable tool for detecting these vascular changes that could act as biomarkers for the severity and progression of this disease. Longitudinal studies in the future are necessary to know more about these vascular alterations and their implications for treatment strategies.

## Introduction

Optical coherence tomography angiography (OCTA) is a noninvasive imaging tool for detailed visualization of the retinal and choroidal microvasculature. It has helped assess various diseases of the retina by giving an exact measurement of the vessel density at the macula. Some studies have described the potential of OCTA in detecting vascular alteration in conditions such as retinitis pigmentosa, age-related macular degeneration, and vitelliform dystrophy [1-3]. These studies indicate that OCTA can highlight macular perfusion deficits, which are crucial for understanding the progression and impact of these retinal conditions. By visualizing the microvascular changes, OCTA provides critical insights that can guide diagnosis, monitoring, and treatment. The ability to detect subtle vascular alterations offers a significant advantage in managing retinal diseases, especially those with a progressive nature [4,5]. Cone dystrophy is a hereditary retinal disorder characterized by severe vision loss because of the progressive degeneration of photoreceptors of cones. Knowing vascular changes in cone dystrophy may help in understanding the pathophysiology and potential targets for the treatment of this disease [6].

This work aims to study the macular vessel density with OCTA in a sample of Iraqi patients with cone dystrophy. We want to contribute to the developing body of understanding that helps form improved diagnosis and treatment strategies for cone dystrophy by analyzing vascular characteristics in these patients.

## Materials and methods

This study was conducted at Gazi Al-Harrir for Surgical Specialty Hospital from July 2024 to 15 of December 2024. The study design was a cross-sectional analysis focusing on the macular vessel density in patients diagnosed with cone dystrophy. Ethical approval was obtained from the hospital's ethical review board (IRB No. 205) in July 2024, and informed consent was secured from all participants before the commencement of the study. Ethical considerations included ensuring the confidentiality and anonymity of all participants, as well as adhering to the principles outlined in the Declaration of Helsinki.

In the present study, inclusion criteria were only clinically diagnosed cases of cone dystrophy, an age range of 10-41 years, and no other significant ocular or systemic conditions that might have altered the retinal vasculature. Healthy subjects of the same age with no history of retinal disease or significant systemic illness were selected as a control group. Exclusion criteria of both groups included previous ocular surgery, significant media opacities, or any condition that might interfere with imaging quality using OCTA. All participants underwent a comprehensive ophthalmic examination, including visual acuity testing, slit-lamp biomicroscopy, and fundus examination. OCTA was performed using a Spectralis OCTA device (Heidelberg Engineering, Heidelberg, Germany). OCTA scans focused on the macular region, capturing the superficial and deep capillary plexus as well as the choriocapillaris. The macular vessel density was measured in the central, superior, inferior, nasal, and temporal subfields. Vascular density was quantified by analyzing the obtained images with built-in software on this device. Steps included in standardization related to imaging proper calibration of the device for acquisition and quality control procedures that assured OCTA measurements were reliable and accurate.

Statistical analysis was performed using Jamovi software (Version 2.3; https://www.jamovi.org/). Descriptive statistics were calculated for all variables, including means, standard deviations, medians, and ranges. The Shapiro-Wilk test was used to assess the normality of the data distributions. Independent samples t-tests were conducted to compare the macular vessel densities between the cone dystrophy group and the control group. The study investigated the effect of gender on macular vessel densities. Descriptive statistics and gender frequencies were calculated, and independent samples t-tests compared the macular vessel densities between male and female participants in both the control and cone dystrophy groups. Correlation analyses were made to examine relationships between age, central retinal thickness, and vessel density in the macular subfields. Ethical considerations included ensuring the confidentiality and anonymity of all participants, and the study adhered to the principles outlined in the Declaration of Helsinki.

## Results

Descriptive statistics

The control group had a mean age of 27.5 years (SD = 4.72), with an equal gender distribution of 50% female and 50% male participants. In contrast, the cone dystrophy group had a mean age of 25.0 years (SD = 12.25), with a predominance of males (75%) compared to females (25%) (Table 1).

**Table 1:** Demographic data of study participants

Demographic Data	Control Group	Cone Dystrophy Group
Total Participants	28	28
Mean Age (years)	27.5 (SD = 4.72)	25.0 (SD = 12.25)
Median Age (IQR)	28 (23.5–31)	23.5 (14–37)
Age Range (years)	21 - 34	10 - 41
Gender Distribution		
- Female	50% (n = 14)	25% (n = 7)
- Male	50% (n = 14)	75% (n = 21)

Macular vessel density

The mean central retinal thickness (CRT) was significantly lower in the cone dystrophy group (180.8 µm) as compared to the control group (238.8 µm). Significant reductions in vessel density were observed in regions in some, but not all, macular regions of cone dystrophy patients as compared to controls: central (13.4 µm vs. 17.7 µm), superior (51.1 µm vs. 48.7 µm), inferior (48.7 µm vs. 46.7 µm), nasal (44.2 µm vs. 43.4 µm), and temporal (45.1 µm vs. 45.5 µm). The Shapiro-Wilk test indicated normal distributions for all parameters except the superior (p = 0.013) and inferior (p < 0.001) fields. Normal distributions were confirmed for these parameters except for the superior field (p = 0.011). Independent samples t-tests for the control group based on Oculus Dexter (OD)/Oculus Sinister (OS) showed no significant differences in the central, superior, inferior, nasal, and temporal fields, with p-values of 0.593, 0.457, 0.184, 0.250, and 0.485, respectively.

Gender differences

When looking into gender differences, it appears there were no major distinctions between males and females across most macular regions in both groups studied. Interestingly, in the control group, females exhibited a notably higher inferior macular vessel density compared to males, with mean values averaging 49.1 (SD = 6.5) for females and 44.3 (SD = 6.5) for males, showing a significant difference (p = 0.008). However, in the cone dystrophy group, no significant gender differences were observed in the central, superior, inferior, nasal, or temporal macular vessel densities.

Correlation analyses

Correlation analyses revealed no significant correlation between age and CRT in the control group (Pearson’s r = 0.948, p = 0.687) and a moderate positive correlation in the cone dystrophy group (Pearson’s r = 0.499, p = 0.208). The correlation between CRT and the superior field in the cone dystrophy group showed a weak negative correlation (Pearson’s r = -0.351, p = 0.183). Linear regression analysis indicated a significant intercept in the cone dystrophy group (p = 0.001), but no significant relationship between CRT and central field thickness. Similarly, in the control group, the intercept was significant (p < 0.001), but no significant relationship was found between CRT and central field thickness. These results indicate significant reductions in macular vessel density in cone dystrophy patients and suggest that while gender differences in macular vessel density are generally not pronounced, there may be specific regional differences in the control group that warrant further investigation (Table 2).

**Table 2:** Associations and gender differences in macular vessel density

Association	Control Group	Cone Dystrophy Group
Correlation between Age and CRT	Pearson’s r = 0.904, p = 0.002	Pearson’s r = 0.499, p = 0.208
Gender Differences in Macular Vessel Density		
- Central	No significant difference, t(6) = 0.539, p = 0.609	No significant difference, t(6) = -1.323, p = 0.234
- Superior	No significant difference, t(6) = -1.211, p = 0.271	No significant difference, t(6) = -0.905, p = 0.400
- Inferior	Significant difference, t(6) = -3.933, p = 0.008	No significant difference, t(6) = -1.384, p = 0.216
- Nasal	No significant difference, t(6) = -0.855, p = 0.425	No significant difference, t(6) = 0.643, p = 0.544
- Temporal	No significant difference, t(6) = 0.024, p = 0.982	No significant difference, t(6) = 1.431, p = 0.202

Effect of age on variables

Correlation analysis revealed a significant positive association between age and nasal-c (r = 0.694, p = 0.056; Spearman’s rho = 0.756, p = 0.030) and inferior-c (r = 0.659, p = 0.075; Spearman’s rho = 0.756, p = 0.030) in the control group. In contrast, the cone dystrophy group showed no significant correlations, except for superior-c (r = -0.578, p = 0.024), which demonstrated a weak negative association with age. These findings suggest a potential age-related influence on specific variables in the control group (Table 3).

**Table 3:** Correlation between age and tested variables in control and cone dystrophy groups

Variable	Control Group (r)	Control Group (p-value)	Cone Dystrophy Group (r)	Cone Dystrophy Group (p-value)
Temporal-c	0.174	0.681	0.128	0.648
Nasal-c	0.694	0.056	0.098	0.727
Inferior-c	0.659	0.075	-0.45	0.093
Superior-c	0.472	0.237	-0.578	0.024
Central-c	0.577	0.134	0.577	0.134

Comparison of retinal macular layers in cases and controls

The comparison of the mean thickness of retinal macular layers between cases and controls revealed a significant difference (Table 4). The inferior-c layer was most affected, with a mean reduction of 4.167 μm in cases compared to controls (p = 0.008, Student's t-test). The nasal-c layer was reduced by 2.117 μm in the cases, but this did not reach statistical significance (p = 0.425). The remaining layers, such as central-c and temporal-c, did not show any significant difference in thickness among the groups in either hemisphere - all p > 0.6.

**Table 4:** Comparison of retinal macular layer thickness between cases and controls

Layer	Mean Difference (Cases - Controls)	SE Difference	p-value (Student's t-test)	p-value (Mann-Whitney U)
Central-c	1.0675	1.981	0.609	0.886
Superior-c	-1.165	0.962	0.271	0.486
Inferior-c	-4.1675	1.06	0.008	0.029
Nasal-c	-2.1175	2.476	0.425	0.343
Temporal-c	0.005	2.32	0.982	0.885

Disc changes in cases and controls

Changes within the optic disc have a statistically significant difference when compared between the cases and the controls. Indeed, all of the cases were noted to have definite changes over the affected side of the optic disc, though such did not appear on controls. To be sure, chi-square demonstrated this association as proper, with χ² = 16.0, df = 1, and p < 0.001. Indeed, these represent strong findings indicating the correlation between changes within the optic disc and conditions in the cases.

## Discussion

In this study, macular vessel density was measured by means of OCTA in Iraqi patients with cone dystrophy. The major finding of this study is the significant decrease in macular vessel density in these patients compared to healthy controls. Particularly, the mean CRT in the control group was 238.8 µm and in the cone dystrophy group, it was significantly lower at 180.8 µm. In addition, vessel density was significantly reduced in all four other regions as compared to controls: the central macular area reduced from 17.7 µm in controls to 13.4 µm in patients, and other regions that had considerable reduction in vessel density were in the superior, inferior, nasal, and temporal regions of the cone dystrophy group.

The results of the current research agree with recent work on several types of retinal disorders. For example, Yılmaz et al. (2021) reported that in patients with cone dystrophy, vessel density was significantly lower compared to healthy controls in most macular areas after studying macular perfusion with the OCTA technique [1]. Similarly, Cennamo et al. (2020) showed low retinal and choriocapillaris vessel density in patients with vitelliform dystrophy, being very useful in the evaluation of macular vascular changes using OCTA [2]. A decrease in vessel density observed in our study corresponds to the vascular changes reported in the above conditions.

In terms of gender comparison, the study found that females had a significantly higher inferior macular vessel density as opposed to males in the control group. However, across all regions of the macula, there were no significant differences between the genders in the cone dystrophy group. This implies that though gender might be responsible for some variations in macular vessel density in normal subjects, cone dystrophy might overlay the difference.

OCTA has been proved a valuable diagnostic tool for the detection of vascular alterations in various diseases of the retina. For example, OCTA was applied to study the macular vascular density in retinitis pigmentosa, which showed distinctively pronounced vascular changes related to the severity of the disease course. In addition, a significant decrease in the densities of deep capillary plexus and choriocapillaris vessels was shown to occur with anti-vascular endothelial growth factor (anti-VEGF) treatment of the neovascular form of age-related macular degeneration. These studies suggest the potential ability of OCTA to identify very subtle changes in vasculature that are particularly important in understanding disease mechanisms and the effects of treatment.

Our results also agree with that of Nakanishi et al., who found the alternations in the photoreceptors of occult macular dystrophy to demonstrate significantly reduced cone densities within the analyzed retinal regions in five cases, using OCT. A possible explanation behind our observation regarding the reduced macular vessel density in this study could be a similar photoreceptor degeneration in cone dystrophies, which also supports the relationship between these vascular changes and the health of the photoreceptors.

The clinical relevance of these findings is high. In this way, OCTA enabled the in-vivo visualization and quantitative assessment of retinal vascular changes, giving significant insight into the pathophysiological mechanisms occurring within the retina in cone dystrophy. Decreased vessel density in the macula could possibly represent a biomarker for the degree of severity and progression of the disease, which would be helpful in its diagnosis and follow-up of patients. Understanding the nature of such vascular alterations helps in devising targeted therapeutic strategies to preserve or restore retinal function.

As for glaucoma studies, it has been reported that OCTA of the macula is able to detect changed vessel density in the superficial and deep vascular plexus of glaucomatous eyes as well [7]. These findings reiterate the diagnostic capability of OCTA in a variety of retinal diseases. The fact that OCTA measurements are further shown to be reproducible and reliable in the pathologic states tested by Lee et al., therefore, further underlines the strength of this imaging modality in such clinical and research applications [8].

In a population of retinitis pigmentosa patients, it was also found that capillary density was reduced compared to healthy controls across all vascular layers [9]. The major decrease in vascular density coupled with major associations of the capillary density with different functions of the retina can once again be mirrored by our findings regarding patients with cone dystrophy.

The work of You et al. (2019) also showed that systemic factors, such as signal strength and axial length, need to be considered when interpreting information derived from OCTA [10]. These were also taken into account in our measurements to assure the reliability and accuracy of our measurements.

Atta-Allah et al. (2018) showed that the OCTA was useful for both the assessment of changes within macular perfusion and the foveal avascular zone in diabetic macular edema (DME) [11]. They found a correlation between reduced vessel density within the deep retinal plexus and decreased VA, which underlined that OCTA-derived biomarkers could be used to foresee the visual outcomes of non-treated diabetic eye in the case of DME. Similarly, Toto et al. (2020) explored the retinal microvascular changes in cone dystrophy patients using OCTA [12]. They found a reduction of both superficial and deep capillary plexus density, positively correlated with the decrease in retinal thickness and functional impairment measured with electrophysiological parameters. This strengthens the important role of OCTA in relating morphological and functional deficits within retinal pathologies.

Lim et al. (2018) pointed out the importance of the strength of the signal for the quantitative assessment of the density of the retinal vascular network using OCTA [13]. They went on to underscore the fact that poor signal strength translates into the gross underestimation of vessel density, perfusion density, and foveal avascular zone metrics. They advised that the minimum signal strength necessary for good quality OCTA metrics should be at least 9 with implications on the interpretation of microvascular changes in different retinal pathologies.

Shahlaee et al. (2016) reported some key normative values of macular vascular density by healthy individuals using OCTA [14]. They demonstrated that the parafoveal vascular density in the deep vascular network was significantly higher compared to the superficial network while the reverse trend was observed in the foveal region. These studies underlined the reproducibility of OCTA regarding the quantification of macular microvascular changes and maybe a non-invasive diagnostic tool for the monitoring of retinal diseases.

The cross-sectional design of this study does not allow any firm judgment on causality or the ability to observe temporal changes in the macular vessel density in cone dystrophy patients. Besides, the generalization of these findings is seriously limited by the sample size. Long-term follow-up of larger cohorts through future studies will be needed to confirm the observations made here and to further elucidate the nature of dynamic vascular changes associated with cone dystrophy.

In conclusion, the current study adds to OCTA's value in detecting and monitoring changes in the retinal vasculature in cone dystrophy. It is important that the large magnitude of reduced macular vessel density in Iraqi cone dystrophy patients be compatible with such magnitude in other retinal disorders. Further longitudinal studies are necessary to evaluate vascular alterations over time and evaluate the impact of potential treatments. In advanced imaging modalities, including OCTA, clinicians can increase their understanding of its implications for managing retinal diseases for improved clinical outcomes.

## Conclusions

This study demonstrated significant reductions in macular vessel density in Iraqi patients with cone dystrophy using OCTA. The findings align with previous research on retinal diseases, highlighting OCTA's utility in detecting subtle vascular changes. These vascular alterations can serve as biomarkers for disease severity and progression, aiding in diagnosis and treatment strategies. Moreover, the study underscores the importance of integrating advanced imaging modalities like OCTA into routine clinical practice, particularly in resource-limited settings, where early detection and monitoring of retinal diseases are crucial. Future research should focus on longitudinal studies to track these changes over time and assess their correlation with functional visual outcomes. Additionally, further studies are needed to explore the potential of OCTA in predicting treatment responses and evaluating therapeutic interventions, ultimately improving patient care and outcomes on a broader scale.
